# Association between neutrophil to lymphocyte ratio and blood glucose level at admission in patients with spontaneous intracerebral hemorrhage

**DOI:** 10.1038/s41598-019-52214-5

**Published:** 2019-10-30

**Authors:** Fan Zhang, Yanming Ren, Wei Fu, Yuelong Wang, Juan Qian, Chuanyuan Tao, Chao You, Mu Yang

**Affiliations:** 1 Department of Neurosurgery, West China Hospital, Sichuan University, Chengdu, China; 2 Department of Pathology, Case Western Reserve University, Ohio, USA; 3 Department of Population and Quantitative Health, School of Medicine, Case Western Reserve University, Ohio, USA; 4 Sichuan Cancer Hospital and Institute, Sichuan Cancer Center, School of Medicine, University of Electronic Science and Technology of China, Chengdu, China; 5 Radiation Oncology Key Laboratory of Sichuan Province, Chengdu, China; 6 Department of Neurology and Neurosurgery, McGill University, Montreal, Canada; 7 Alan Edwards Centre for Research on Pain, McGill University, Montreal, Canada

**Keywords:** Stroke, Stroke, Risk factors, Risk factors

## Abstract

Previous studies indicated that both inflammatory responses and hyperglycemia are involved in the similar pathophysiological mechanisms after onset of intracerebral hemorrhage (ICH). However the relationship between hyperglycemia and inflammation remains unknown. We aim to evaluate the associations of hyperglycemia with inflammation and neutrophil to lymphocyte ratio (NLR) in patients with ICH. Patients with acute ICH were retrospectively enrolled. Clinical characteristics and imaging features were obtained. The associations between outcome and laboratory biomarkers were assessed by multivariable logistic regression analysis. Spearman analysis and multiple linear regression analysis were performed to estimate the association of NLR and serum glucose. 175 patients were enrolled. Poor outcome occurred in 86 patients at 30 days. Elevated blood glucose level (BGL) and NLR were strongly associated with outcome in patients with ICH. Moreover, combined NLR-BGL exhibited a better predictive accuracy compared with the peripheral leukocyte counts. Furthermore, there was a robust association between BGL and NLR. We first demonstrated both of NLR and BGL were independently associated with each other. Our results indicate that inflammatory responses and the pathological process of hyperglycemia may influence each other by several complex pathological mechanisms and have a mutual promoting effect to secondary brain injury.

## Introduction

Spontaneous intracerebral hemorrhage (sICH, also known as ICH) is a devastating healthcare event accounting for 10–15% of all strokes^1,2^. It carries high mortality and morbidity with limited efficient therapeutic treatment^3^. Hyperglycemia and inflammatory responses were widely recognized to play a crucial role in the pathophysiological mechanism of ICH and lead to secondary brain injury^4–7^. And it is well-known that both hyperglycemia and inflammatory response contribute to the similar pathological progression in ICH, such as hematoma expansion^8–10^ or brain edema^11–13^. Furthermore, the experiment studies^14–16^ have revealed there was a connection in the pathophysiological mechanisms between high serum glucose level and inflammatory activity. However the relationship between hyperglycemia and inflammation in ICH patients has not been reported. The aim of this report is to estimate the association between hyperglycemia and inflammation in the ICH cohort, as well as a novel inflammatory marker known as neutrophil to lymphocyte ratio (NLR), which was recently reported to be identified as an ideal inflammatory predictor for functional outcome in ICH patients^9^.

## Methods

### Patient selection

We retrospectively identified all the patients with spontaneous intracranial hemorrhage visited to West China hospital from February 2017 to December 2017. We defined inclusion criteria as follows: (1) A diagnosis of intracranial hemorrhage by computed tomography (CT); (2) Blood routine examination and laboratorial tests were conducted within 24 hours after ictus; (3) ≥ 18 years. We excluded patients due to: (1) that ICH was attributable to trauma, brain neoplasm, aneurysm or arteriovenous malformation; (2) that a history of infection or obvious sign of acute/chronic infection was observed within 2 weeks; (3) unavailable CT scans or follow-up data; (2) systemic diseases including lung cancer, chronic obstructive pulmonary disease, tuberculosis, autoimmune disease, severe renal dysfunction and so on; (4) consumption of immunosuppressant drug or anticoagulants; (5) a stroke history within 6 months displayed in the electric medical record.

### Ethical approval and informed consent

This study was approved by the Biomedical Ethic Committee at Sichuan University and all patients’ legal surrogates have given their informed consent. This study was also conducted in accordance with relevant guidelines and regulations of Sichuan University.

### Clinical and radiological data

The demographic and clinical characteristics were collected at hospital arrival, including age, sex, blood pressure, cigarette consumption and alcohol use, medical history of hypertension and diabetes mellitus, history of ischemia stroke and aneurysm. Laboratorial variables were also recorded including blood glucose level (BGL), white blood cells (WBC), absolute neutrophil count (ANC), absolute lymphocyte count (ALC), absolute monocyte count (AMC). The laboratorial data were collected from blood biochemical test and admission blood work which were conducted on the automated hematology analyzer KX-21 N (Sysmex America, Inc., Lincolnshire, Illinois, USA). Neutrophil to lymphocyte ratio (NLR) was calculated as absolute neutrophil count (ANC) divided by absolute lymphocyte count (ALC). Combined NLR-BGL score was calculated as the total scores of NLR and BGL. Patients with increased NLR (>cut-off threshold) were given a score of 1, increased BGL a score of 1. The functional outcome was assessed by 30-day Glasgow Outcome Scale (GOS) scores by telephone or outpatient visiting. GOS ≤3 was defined as unfavorable outcome including severe disability, persistent vegetative state, as well as death.

Two reviewers, who were blinded to clinical conditions of patients, independently read and estimated all the head CT scans. Any disagreement between the two neuroradiologists was solved by the consensus. Radiological results collected from head CT within 24 h from ictus included hematoma location, hematoma size, presence of subarachnoid hemorrhage, intraventricular hemorrhage, cerebral infarction and hydrocephalus. Hematoma volume was measured by ABC/2 method as described previously^17^.

The datasets analysed during the current study are available from the corresponding author on reasonable request.

### Statistical analysis

All the baseline characteristics including clinical variables, laboratorial parameters and radiological data were compared between patients with poor outcome and with favorable outcome. Continuous variables were expressed as mean ± standard deviation or median with interquartile range (IQR) for normal distribution and non-normal distribution, respectively, whereas categorical variables were expressed as frequency and percentage. Univariate analyses were conducted by independent t test or Mann–Whitney U test or Chi-square (χ^2^) test or Fisher’s exact test. Independent t test or Mann–Whitney U test were applied to compare continuous variables. Chi-square (χ^2^) test or Fisher’s exact test were conducted to compare categorical data. The variables which exhibited a statistically significant difference (P < 0.05) in univariate analysis were then forced into multivariable logistic regression analysis. Spearman analyses were preformed to estimate the correlations of BGL or NLR with other clinical variables. The variables presenting significant relationships (P < 0.05 in Spearman analysis) with BGL/NLR were then included into multiple linear regression analysis to assess the association of these factors with BGL/NLR. Receiver-operator analysis was applied to estimate the predictive ability of NLR and BGL and combined NLR-BGL for short-term prognosis of ICH patients. The variables with a P value of <0.05 were considered significant. All the above-mentioned statistical analyses were determined by applying SPSS 23.0.

## Results

**Figure 1:**
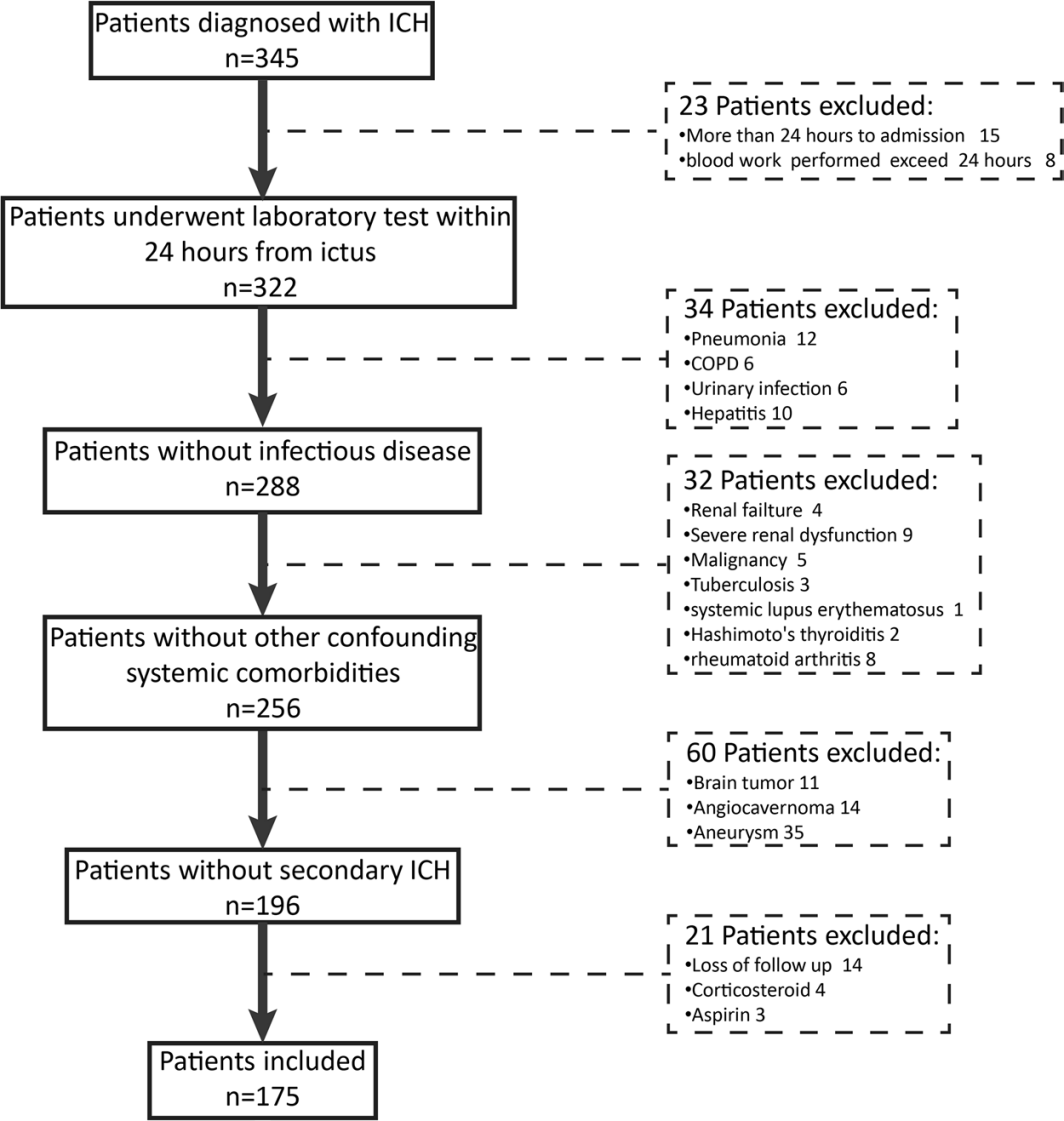
Flowchart of patient enrollment. ICH intracerebral hemorrhage; COPD chronic obstructive pulmonary disease.

From February 2017 to December 2017, 175 consecutive patients (124 males and 51 females) with spontaneous ICH fulfilling the inclusive criteria were enrolled in this retrospective study (Detail exclusion of patients in Fig. 1). The mean age was 60.06 ± 13.01 years ranging from 32 to 93 years. The mean baseline hematoma size was 28.82 ± 16.56 ml and the median GCS (Glasgow Coma Scale) score at hospital arrival was 11 with IQR of 8 to 14. 114 (65.1%) patients had a history of hypertension, 12 of diabetes mellitus, 29 of cerebral infarction. The 1-month mortality was 25.1% (44 patients). 86 patients had an unfavorable outcome defined as a GOS of ≤3 at 30 days. Infratentorial hemorrhage occurred in 18 patients in which 10 individuals (55.6%) with poor outcome were found. By comparison, 76 patient (48.4%) with supratentorial hemorrhage had unfavorable outcome (P = 0.56). Table 1 showed the main clinical characteristics and radiological finding.

**Table 1:** Clinical characteristics related to 30-day outcome in patients with ICH.

Characteristic	Baseline n = 175	favorable outcome (n = 89)	poor outcome (n = 86)	P
Male	124(70.9)	64(71.9)	60(69.8)	0.76
Age(years)	60.06 ± 13.01	58.56 ± 10.74	61.62 ± 14.90	0.12
Systolic blood pressure(mmHg)	165.18 ± 30.47	164.85 ± 32.39	165.52 ± 28.53	0.89
Diastolic blood pressure(mmHg)	94.09 ± 19.71	95.58 ± 19.42	92.56 ± 19.99	0.31
Mean arterial pressure(mmHg)	117.47 ± 21.76	118.28 ± 22.84	116.63 ± 20.69	0.62
Medical history	—	—	—	—
Hypertension	114(65.1)	59(66.3)	55(64.0)	0.75
Diabetes mellitus	12(6.9)	7(7.9)	5(5.8)	0.59
Cerebral infarction	29(16.6)	13(14.6)	16(18.6)	0.48
Smoking	72(41.1)	37(41.6)	35(40.7)	0.91
Drinking	64(36.6)	34(38.2)	30(34.9)	0.65
GCS score on admission	11(8, 14)	14(11,15)	8(6, 10)	<0.01*
Hematoma size (ml)	28.82 ± 16.56	20.06 ± 10.74	37.88 ± 16.70	<0.01*
Presence of SAH	34(19.4)	16(18.0)	18(20.9)	0.62
Presence of IVH	44(25.1)	12(13.5)	32(37.2)	<0.01*
Infratentorial hematoma	18(10.3)	8(9.0)	10(11.6)	0.57
Hydrocephalus	31(17.1)	12(13.5)	19(22.1)	0.14
BGL mmol/L	7.93 ± 2.94	6.77 ± 1.95	9.12 ± 3.31	<0.01*
WBC 10^9^/L	12.21 ± 4.44	10.73 ± 4.14	13.74 ± 4.25	<0.01*
ANC 10^9^/L	10.12 ± 4.38	8.68 ± 4.12	11.60 ± 4.15	<0.01*
ALC 10^9^/L	1.23 ± 0.81	1.28 ± 0.59	1.17 ± 0.98	0.39
AMC 10^9^/L	0.56 ± 0.28	0.55 ± 0.31	0.58 ± 0.25	0.61
NLR	9.37(5.95, 14.42)	7.61(3.94, 12.78)	10.95(8.39, 16.46)	<0.01*
NLR-BGL	2(1, 2)	1(0, 2)	2(1.75, 2)	<0.01*

Data are expressed as n(%), mean ± standard deviation, median(interquartile range), as appropriate.*P < 0.05.

SAH, subarachnoid hemorrhage;IVH, intraventricular hemorrhage; GCS, Glasgow coma scale; WBC, white blood cells; ANC, admission neutrophil count; ALC, admission lymphocyte count; AMC, admission monocyte count; NLR, neutrophil to lymphocyte ratio; BGL, blood glucose level.

The patients with poor outcome at 30 days had higher blood glucose level (BGL), white blood count (WBC), absolute neutrophil count (ANC), neutrophil to lymphocyte ratio (NLR) and lower GCS scores whereas no difference was found in the absolute lymphocyte count (ALC) or absolute monocyte count (AMC) (Table 1). Univariate analysis found blood glucose level (BGL), WBC, ANC and NLR was associated with unfavorable outcome (Table 2). However, after adjustment for potential confounding clinical variables, multivariable analysis demonstrated only BGL (OR: 1.34; 95% CI: 1.05–1.72; p = 0.02) and NLR (OR: 1.09; 95% CI: 1.03–1.17; p = 0.03) were independently predictive of 1-month functional outcome (Table 2).

**Table 2:** Associations of clinical characteristics and laboratory values on admission with 30-day poor outcome in patients with ICH.

Characteristic	Crude		Adjusted	
	OR (CI)	P	OR (CI)	P
Serum glucose level mmol/L	1.549 (1.289–1.861)	<0.01	1.341 (1.045–1.720)	0.02*
WBC 10^9^/L	1.182 (1.095–1.276)	<0.01	1.032 (0.925–1.153)	0.57
ANC 10^9^/L	1.194 (1.094–1.327)	<0.01	1.033 (0.921–1.160)	0.59
NLR	1.059 (1.021–1.099)	<0.01	1.085 (1.025–1.168)	0.03*
NLR-BGL	4.878 (2.862–8.315)	<0.01	2.238 (1.123–4.266)	0.01*

CI, confidence interval; OR, odds ratio; WBC, white blood cells; ANC, admission neutrophil count; NLR, neutrophil to lymphocyte ratio; BGL, blood glucose level.

Adjustment of confounding factors on admission with 30-day poor outcome were determined by a logistic regression analysis using SPSS 23.0. The confounding factors include GCS score, hematoma volume and presence of IVH. *Indicates P < 0.05.

**Figure 2:**
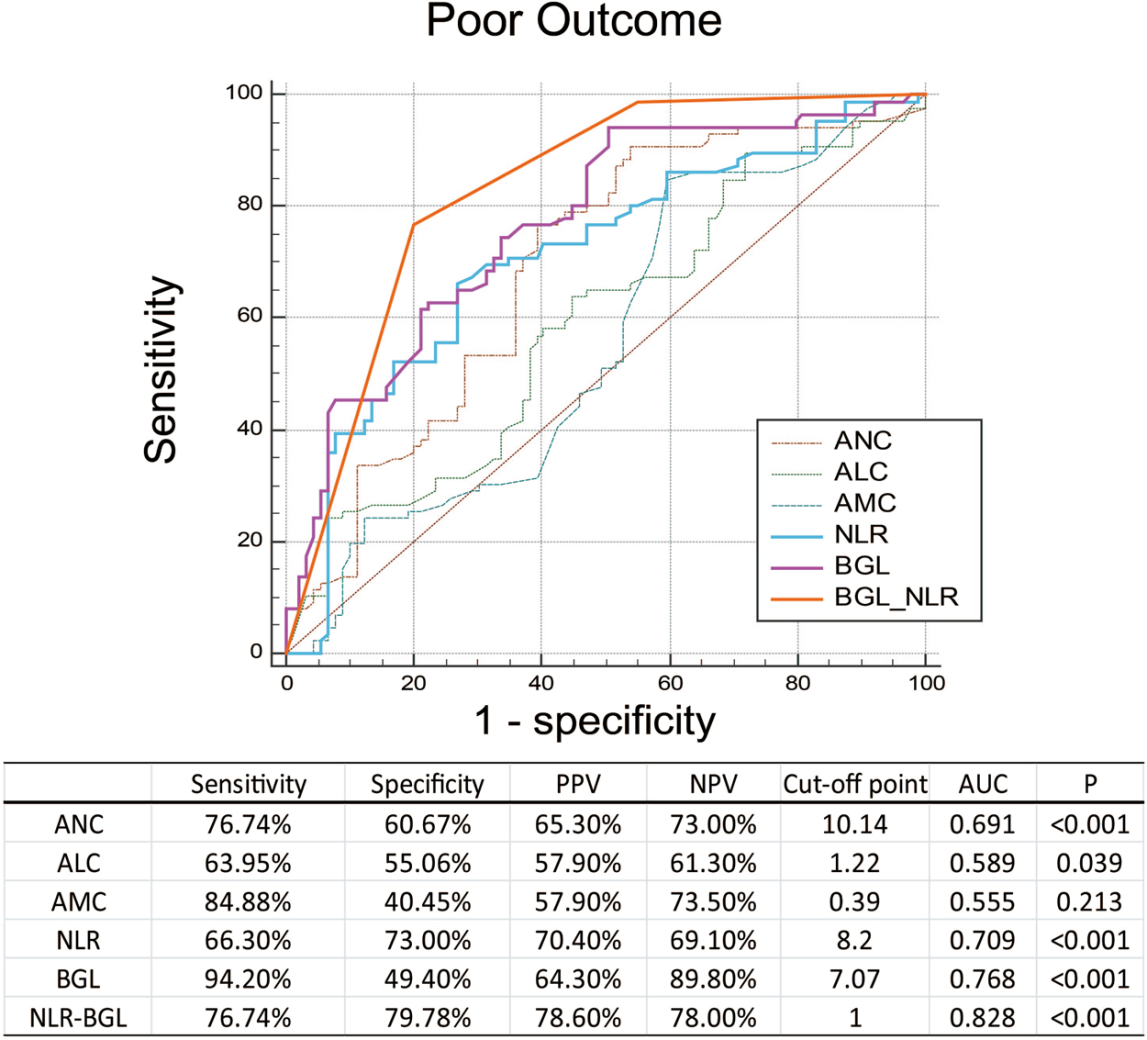
Receiver operating characteristic curves of ANC, ALC, AMC, NLR, BGL, NLR-BGL with sensitivity, specificity, positive predictive value (PPV) and negative predictive value (NPV), areas under the curve (AUC) for predicting 90-day unfavorable outcome in ICH patients. ANC, admission neutrophil count; ALC, admission lymphocyte count; AMC, admission monocyte count; NLR, neutrophil to lymphocyte ratio; BGL, blood glucose level.

The receiver operating characteristic analysis (ROC) was performed to compare the predictive ability of relevant biomarkers for the short-term prognosis of ICH patients. The sensitivity, specificity, positive predictive value (PPV), negative predictive value (NPV) and the area under the curve (AUC) of NLR for prognostic prediction were 66.30%, 73.00%, 70.40%, 69.10% and 0.709, respectively. And the serum glucose exhibited a comparable AUC of 0.768 to that of NLR. We further combined these independent predictors as NLR-BGL to evaluate its predictive ability in 30-day functional outcome of ICH patients. Without surprise, NLR-BGL was significantly associated with the 30-day poor outcomes in the univariate and multivariate analysis (OR: 2.238; 95% CI: 1.123–4.27; p < 0.01) (Tables 1 and 2). Figure 2 shows the ROC curves and the areas under the curves (AUC) of laboratory parameters for predicting poor outcome. The combined NLR-BGL showed the best predictive value with AUC 0.828 when setting a score of 1 as the cut-off point (sensitivity 76.74%, specificity 79.78%).

Spearman’s analysis demonstrated NLR and BGL were all correlated with age, smoking, admission GCS scores, hematoma volume, WBC, ANC, ALC (Table 3). Multiple linear regression analysis were then applied and identified GCS at admission, hematoma size and ANC were linearly correlated with both of NLR (Table 4) and BGL (Table 5). This analysis also demonstrated that supratentorial hemorrhage, WBC, ALC were independently predictive of NLR (Table 4) while diabetes and IVH can predict BGL (Table 5). Moreover, we found that both NLR and BGL were associated with each other for first time (Tables 4 and 5).

**Table 3:** Spearman correlation analysis with neutrophil to lymphocyte ratio and admission blood glucose in patients with ICH.

Characteristics	NLR		BGL	
	r	P	r	P
Male	−0.026	0.731	−0.07	0.375
Age(years)	0.189	0.012*	0.287	<0.001*
Systolic blood pressure(mmHg)	0.269	<0.001*	−0.011	0.881
Diastolic blood pressure(mmHg)	0.116	0.126	−0.156	0.039*
Mean arterial pressure (mmHg)	0.179	0.018*	−0.126	0.096
Hypertension	−0.016	0.835	0.129	0.088
Diabetes mellitus	−0.147	0.16	0.204	0.004*
Ischemic stroke	0.09	0.238	0.086	0.258
Smoker	−0.167	0.027*	−0.155	0.041*
Alcohol (>3 drinks per 24 hours)	−0.042	0.577	0.002	0.978
GCS score on admission	−0.384	<0.001*	−0.361	<0.001*
Hematoma size (ml)	0.341	<0.001*	0.329	<0.001*
Presence of SAH	0.069	0.362	0.113	0.138
Presence of IVH	0.145	0.056	0.256	0.001*
Hydrocephalus	−0.031	0.68	0.064	0.401
Infratentorial hematoma	−0.215	0.004*	0.06	0.433
WBC	0.578	<0.001*	0.335	<0.001*
ANC	0.677	<0.001*	0.383	<0.001*
ALC	−0.789	<0.001*	−0.304	<0.001*
AMC	−0.029	0.707	0.054	0.477
NLR	—	—	0.461	<0.001*
Serum glucose	0.461	<0.001*	—	—
GOS at 90 days	−0.309	<0.001*	−0.426	<0.001*

Asterisks (*) indicate P value < 0.05.

SAH, subarachnoid hemorrhage; IVH, intraventricular hemorrhage; GCS, Glasgow coma scale; WBC, white blood cells; ANC, admission neutrophil count; ALC, admission lymphocyte count; AMC, admission monocyte count; NLR, neutrophil to lymphocyte ratio; BGL, blood glucose level.

**Table 4:** Multivariable linear regression with neutrophil to lymphocyte ratio.

Characteristic	NLR	
	Standardized coefficient	P
GCS score on admission	−0.278	0.026*
Hematoma size	0.302	0.006*
Infratentorial hematoma	−0.181	0.004*
WBC	1.108	<0.001*
ANC	2.383	<0.001*
ALC	−0.893	<0.001*
BGL	0.318	0.027*

Adjustment of confounding factors on NLR were determined by multiple linear regression analysis using SPSS 23.0. The confounding factors include age, smoking, GCS score on admission, hematoma size, systolic blood pressure, mean arterial pressure, infratentorial hematoma, WBC, ANC, ALC and BGL.

GCS, Glasgow coma scale; WBC, white blood cells; ANC, admission neutrophil count; ALC, admission lymphocyte count; AMC, admission monocyte count; NLR, neutrophil to lymphocyte ratio; BGL, blood glucose level. Asterisks (*) indicate P value < 0.05.

**Table 5:** Multivariable linear regression with serum blood level.

Characteristic	BGL	
	Standardized coefficient	P
Diabetes mellitus	0.622	<0.001*
GCS score on admission	−0.331	<0.001*
Hematoma size	0.241	<0.001*
Presence of IVH	0.252	0.001*
ANC	0.166	0.029*
NLR	0.183	0.013*

Adjustment of confounding factors on BGL were determined by multiple linear regression analysis using SPSS 23.0. The confounding factors include age, smoking, GCS score on admission, hematoma size, diastolic blood pressure, diabetes mellitus, IVH, WBC, ANC, ALC and NLR.

GCS, Glasgow coma scale;ANC, admission neutrophil count; NLR, neutrophil to lymphocyte ratio; BGL, blood glucose level. Asterisks (*) indicate P value < 0.05.

## Discussion

The study systematically investigated the impact of NLR and glucose on 30-day poor outcome of patients with ICH. The major findings in the patients with ICH included: (1) elevated NLR and serum glucose can independently and negatively influence 30-day outcome; (2) combined NLR and glucose (as NLR-BGL) can better predict the unfavorable outcome than any other biomarkers alone; (3) admission GCS scores, hematoma volume and neutrophil counts independently predict both NLR and serum glucose; (4) NLR can predict BGL independently and BGL is also strongly predictive of NLR. Moreover, the critical thresholds of 7.07 for glucose, 8.20 for NLR were identified to be best at predicting 30-day outcome of ICH. To the best of our knowledge, this is the first research to estimate the association between NLR and serum glucose in ICH in the clinical observational trial. The composite effect of NLR and glucose in the setting of ICH is also assessed for the first time.

The predictive values of inflammation and hyperglycemia after ICH capture tremendous attentions for past two decades since both inflammatory response and increased blood glucose level are potentially preventable. Numerous evidence showed hyperglycemia^18–20^ and inflammation^9,21,22^ were associated with adverse outcome in patients with ICH. Bejot Y^23^ demonstrated that elevated admission blood glucose independently predicted 1-month fatality rate and poor functional outcome at discharge from a France population-based study, while Fogelholm R^24^ reported admission hyperglycemia was associated with early death regardless of diabetic status in Finland population. On the other hand, Suzuki S *et al*.^25^ found higher leukocyte counts were associated with larger hematoma size. Leira R *et al*. and Sun W *et al*.^26,27^ further reported increased leukocyte level and lower ALC/lymphocytopenia could independently predict early neurologic deterioration and 1-month poor outcome in ICH patients, supporting that the leukocyte play an important role in ICH. However, presence of acute or chronic infections at admission which obviously influenced leukocyte and its subtypes, were not excluded in these studies. Potential confounding factors including systematic comorbidities which may impact the inflammatory response and blood level were not excluded neither. This current study with strict inclusion criteria revealed that patients with unfavorable outcome at 1 month had significantly higher WBC, ANC, BGL and NLR, providing a convincing evidence for these associations of inflammation and serum blood on the prognosis of ICH, but we failed to found independent associations of ANC and leukocyte on poor outcome. Only BGL and NLR remained independently correlated with poor outcome after correction for confounding factors.

NLR emerged as a novel systemic inflammatory predictor in the stroke studies^28,29^. It has been reported NLR was associated with short-term and long-term mortality in patients after ischemic stroke independent from ischemic size^30,31^. NLR had also been identified as a better independent predictor in ICH patients for 30-day mortality^22^ and 90-day mortality^32^ and unfavorable outcome^9^. It is easily calculated and represents a readily available routine makers as well as glucose. Moreover, NLR had a comparable predictive value to BGL for the 30-day outcome in ICH patients according to our ROC analysis. However, the combined biomarker (BGL-NLR) exhibited a better predictive value for ICH patients than any other index. This may be due to the delicately different roles they played in the ICH. NLR reflected the information with regard to systematic inflammation while the high blood glucose at admission represented a stress response level and involved in several molecular mechanisms inducing brain edema, mitochondria disruption and hematoma expansion^12,23^, finally triggered a complex hyperglycemia-induced brain injury. Considering that inflammation, ischemia, brain edema and hematoma enlargement played crucial roles in pathophysiological process after onset of ICH, combined NLR-BGL represented the strongest predictor after ICH in this research should be reasonable.

The pathological mechanisms of adverse effect of hyperglycemia on outcomes after ICH involving oxygen-free radical generation^33^ and the blood-brain barrier breakdown^34,35^, were similar to the pathophysiological processes of inflammatory in experimental study^12,14–16,36^. However, the association between inflammation and hyperglycemia had never been investigated in the clinical study. The present research conducted a multiple linear regression analysis and demonstrated that NLR and BGL had a robust association with each other in an ICH patient cohort for the first time, consistent with these laboratory findings. The plausible explanations for this connection are stated as following. First, inflammation was proposed to rapidly impact the metabolic status after onset of ICH including glucose level^6,37,38^, indicating the inflammatory response could induced the hyperglycemia. Second, studies had demonstrated the deleterious effect of increased blood glucose accelerated blood-brain barrier damage, impaired microvascular integrity and promoted continuous bleeding^39,40^, which could generate thrombin to activate the inflammatory cascade resulting in elevated white blood cell count, C-reactive protein and interleukin-6^41,42^. Hyperglycemia could also increase inflammatory cytokine (including tumor necrosis factor-α (TNF-α) and interleukin-18 (IL-18)) by a oxidative stress mechanism in human^15^, suggesting hyperglycemia could produce an inflammatory response. On the other hand, animal experiment manifested hyperglycemia could increase TNF-α and IL-1b in ICH rats^43^. In addition, both of hyperglycemia and inflammation were widely accepted to contribute to the same pathophysiological progression of ICH including hematoma expansion^8–10,25,44,45^ and brain edema^11,12^. And both of them were supposed to be the indirect signs of stress resulted from ICH^6^. Therefore, we believe the inflammatory responses and the pathophysiological mechanism of hyperglycemia impact each other through several complex signal pathways and altogether produce a mutual promotion effect to secondary brain injury, though the underlying signal pathways are still unclear. Further study was urgently needed to be performed to clarify the definite biological mechanisms.

Several limitations should be taken into accounts when interpreting our results. First, all the patients enrolled in this study came from a single institution-West China hospital, which is a large comprehensive hospital in China. The patients may present with worse clinical grades compared with other hospitals owing to the medical referral system according to Chinese health policy. Second, a relatively large proportion of patients excluded due to the strict inclusive criterion or missing laboratory parameter may result in a selection bias. Third, this study is a retrospective clinical research and the sample size is relatively small. Finally, ABC/2 method for hematoma size was less accurate than modern planimetric techniques.

## Conclusions

Our study first demonstrated there is a strong association between NLR and hyperglycemia in clinical study. This current study also confirmed that NLR and blood glucose level can independent predicted 30-day functional outcome and first revealed that combined index NLR-BGL exhibited a better predictive accuracy than any other biomarker alone. These findings indicated that inflammatory responses and hyperglycemia can impact each other through multiple signal pathways and had a mutual deleterious effect for the short-term prognosis of patients with ICH. Further studies are urgently needed to investigate the exact biological mechanisms.
